# Accuracy and Reliability of Pallor for Detecting Anaemia: A Hospital-Based Diagnostic Accuracy Study

**DOI:** 10.1371/journal.pone.0008545

**Published:** 2010-01-01

**Authors:** Ashwini Kalantri, Mandar Karambelkar, Rajnish Joshi, Shriprakash Kalantri, Ulhas Jajoo

**Affiliations:** Department of Medicine, Mahatma Gandhi Institute of Medical Sciences, Wardha, Maharashtra, India; Universidad Peruana Cayetano Heredia, Peru

## Abstract

**Background:**

Anaemia is a common disorder. Most health providers in resource poor settings rely on physical signs to diagnose anaemia. We aimed to determine the diagnostic accuracy of pallor for anaemia by using haemoglobin as the reference standard.

**Methodology/Principal Findings:**

In May 2007, we enrolled consecutive patients over 12 years of age, able to consent and willing to participate and who had a haemoglobin measurement taken within a day of assessment of clinical pallor from outpatient and medicine inpatient department of a teaching hospital. We did a blind and independent comparison of physical signs (examination of conjunctivae, tongue, palms and nailbed for pallor) and the reference standard (haemoglobin estimation by an electronic cell counter). Diagnostic accuracy was measured by calculating likelihood ratio values and 95% confidence intervals (CI) at different haemoglobin thresholds and area under the receiver operating characteristic curve. Two observers examined a subset of patients (n = 128) to determine the inter-observer agreement, calculated by kappa statistics. We studied 390 patients (mean age 40.1 [SD 17.08] years); of whom 48% were women. The haemoglobin was <7 g/dL in 8% (95% confidence interval, 5, 10) patients; <9 g/dL in 21% (17, 26) patients and <12 g/dL in 64% (60, 70) patients. Among patients with haemoglobin <7 g/dL, presence of severe tongue pallor yielded a LR of 9.87 (2.81, 34.6) and its absence yielded a LR of 0. The tongue pallor outperformed other pallor sites and was also the best discriminator of anaemia at haemoglobin thresholds of 7 g/dL and 9 g/dL (area under the receiver operating characteristic curves (ROC area  = 0.84 [0.77, 0.90] and 0.71[0.64, 0.76]) respectively. The agreement between the two observers for detection of anaemia was poor (kappa values  = 0.07 for conjunctival pallor and 0.20 for tongue pallor).

**Conclusions/Significance:**

Clinical assessment of pallor can rule out and modestly rule in severe anaemia.

## Introduction

Anaemia is a common disorder, affecting a third of the world population most of whom live in resource poor countries [1]. Although diagnosis of anaemia can easily be done by traditional Sahli's haemoglobinometer, or more recently by electronic cell counters, yet physicians and healthcare workers try to detect anaemia by looking at conjunctival, tongue, palmer, or nailbed pallor [2]. Often physicians use clinical assessment of pallor as a screening test, and order haemoglobin test if one or more sites suggest presence of pallor. This is especially true of crowded outpatients departments of public hospitals, where most doctors either believe that accurate estimation of haemoglobin is either not worth the time and effort needed to obtain it or do not have access to facilities to measure haemoglobin.

The physical signs to diagnose anaemia include conjunctival, tongue, palmer, and nailbed pallor [2]. Diagnostic studies assessing the accuracy of pallor for detection of anaemia have largely focused paediatric population. According to a systematic review on the accuracy of clinical signs of anaemia [3] which included 11 studies (8726 children), mostly performed in Africa, the rates of false positive and false negative results were unacceptably high for the clinical diagnosis of anaemia. In the four diagnostic studies that evaluated the accuracy of pallor in the conjunctivae, face, palms and nailbeds to detect anaemia in adult inpatients, the sensitivity and specificity of pallor ranged from 19 to 70 percent and 70 to 100 percent respectively [2], [4], [5], [6].

Although the clinical signs for detection of anaemia are imperfect, these signs can be easily elicited at the bedside, with little training. Looking for pallor is deeply embedded in clinical teaching and physical examination, and despite limitations, this practice is unlikely to be discarded. It is important for health-care workers to know the accuracy of pallor in detecting anaemia, and if positive what level of anaemia clinical pallor can detect with confidence. The aim of the present study was to determine accuracy and reliability of clinical pallor to detect moderate and severe anaemia among patients aged 12 years or more, presenting to inpatient or outpatient departments of a teaching hospital. We also aimed to compare accuracy across different sites, to know assessment of which site has highest accuracy.

## Methods

### Ethics

The study was approved by the ethics committee of Mahatma Gandhi Institute of Medical Sciences (IRB00003623). We obtained a written informed consent from all study participants before enrolling them in the study.

### Setting

The Mahatma Gandhi Institute of Medical Sciences, Sevagram is a 620-bed teaching hospital located in a town in Central India. In outpatient setting, physicians typically order haemoglobin estimation in patients clinically judged to have anaemia, in all women who come for antenatal assessment, or as part of preoperative assessment. On the other hand almost all patients admitted to the hospital undergo haemoglobin estimation. According to our hospital information system (unpublished data), of the 10460 patients discharged from the medicine wards in 2007, 515 (4.9%) were assigned International Classification of Diseases (ICD-10) codes of anaemia. We enrolled patients from both outpatient and inpatient settings in this study.

### Study Design

Between 1 May and 15 May 2007, we prospectively studied all consecutive medicine inpatients. Between 22 May and 29 May, 2007, we also studied all outpatients who visited a central diagnostic laboratory for complete blood counts. We excluded patients if we knew their haemoglobin values before we examined them, or if they were bleeding or receiving a blood transfusion at the time of enrolment. It is a common cultural practice for women to embellish their palms with *mehendi* or apply *kajal* on their conjunctivae. We excluded these pallor sites from the final analyses.

### Index Tests

After obtaining an informed consent, and blind to the diagnosis, physical findings and laboratory data, an observer (AK) sequentially examined conjunctiva, tongue, palm and nailbed of each study patient. Pallor at any site was classified as being absent, mild, moderate or severe. The conjunctiva was considered pale, if the anterior rim of the lower palpebral conjunctiva looked as pale as the deeper posterior rim. The tongue pallor was assessed on the dorsum of the tongue. Palmer pallor was assessed by the intensity of the colour of the palmer creases. Nailbed pallor was assessed by the colour of the nail.

To assess inter-observer variability in the interpretation of pallor, two observers, AK and MK (years of graduation, 2007 and 2009 respectively) independently examined the four pallor sites. The interval between the two observer's examinations ranged between 15 minutes and 3 hours.

### Reference Standard

The reference standard was haemoglobin estimated by an electronic cell counter (Beckman Coulter, Inc. Fullerton, CA, USA) in the haematology division of the central laboratory. The period between the clinical examination and the haemoglobin estimation did not exceed 24 hours. The technician performed the test within 2 hours of blood withdrawal. We used the hospital information system to retrieve the test results of all study patients, after the data collection for the study was over.

### Sample Size

In our study, we assumed the estimates of sensitivity and specificity for pallor to be 70% each, based on the published literature. We focused our sample size calculation on target- positive patients and calculated that to achieve 95% confidence interval (CI) ±10% of estimates of sensitivity and specificity; we needed to recruit 78 target- positive patients. In our setting, the prevalence of haemoglobin <9 g/dL is 20% (Unpublished data, MGIMS hospital information system). We therefore enrolled 390 patients.

### Statistical Analysis

The observers used an ordinal scale to judge pallor (no, mild, moderate and severe). We also used, *a priori*, three cutoff points for haemoglobin (7 g/dL, 9 g/dL and 12 g/dL) to classify anaemia as “severe”, “moderate” and “mild” and to assess the accuracy of palmer at different haemoglobin thresholds. We compared mean haemoglobin levels at each haemoglobin cutoff point using student's *t*-test. P values <0.05 were considered significant. We calculated likelihood ratios (LR) and their 95% confidence intervals at different levels of pallor. LR is a likelihood of a test result in a person with disease compared with the likelihood of this result in a person without disease. LRs close to 0 virtually rule out disease, while LRs bigger than10 almost rule in disease.

To determine ability of different index tests to distinguish patients with and without target disorder, we compared their areas under the receiver operating characteristic (ROC) curves. ROC curve is a plot of sensitivity of test (Y-axis) against 1-specificity (X-axis). The closer the curve gets to the upper left corner of the graph, larger is the area under the curve (AUC) and better is the accuracy of the test. Values close to 1 indicate a very informative test; values close to 0.5 indicate an uninformative test.

We measured the inter-observer variability in the interpretation of pallor among the two observers by kappa (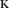 ) statistic, a measure of agreement corrected for chance. A 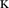 value of 0 indicates that the observed agreement is same as would be found by chance. We used the following guidelines to interpret 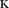 statistic: <0.2, poor agreement; 0.2–0.4; fair agreement; 0.4–06, moderate agreement; 0.6–0.8, good agreement; and 0.8–1.0, excellent agreement [7].

All analyses were done with Stata software (Stata 10, Stata Corporation, Texas, USA).

## Results

**Figure 1:**
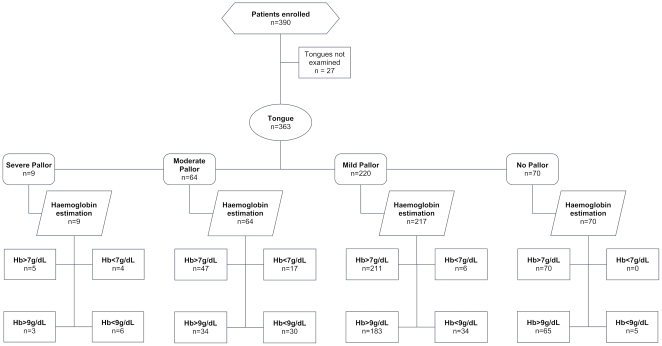
Sample Study Profile, tongue pallor as index test and haemoglobin levels (less than 7 g/dL and 9 g/dL) as reference standards.

We used STARD (Standards for Reporting Diagnostic Accuracy) statement to report this study. Figure 1 shows the sample study profile. In May 2007, a total of 390 patients, 12 years of age and older were enrolled in the study (mean age 40.1 [SD] 17 years, range: 12 to 87 years; 190 [48%] females). Of the 390 patients, 208 were medical inpatients and 182 were outpatients. AK (observer 1) examined a total of 390 patients of whom MP (observer 2) examined 128 patients. The observer 1 could not interpret pallor on six conjunctivae, 27 tongues, 8 palms and 15 nailbeds. We could not measure haemoglobin in 3 patients.

The mean haemoglobin of the study population was 11.1 (SD 2.6) g/dL. Twenty-eight patients (7%) had severe anaemia; 55(14%) had moderate anaemia; 170(44%) had mild anaemia and 134 (35%) patients were non-anaemic. The mean haemoglobin levels in the inpatients and the outpatients were 10.80 g/dL and 11.32 g/dL respectively (p = 0.04). Except nailbed, the mean haemoglobin levels were significantly different across the pallor grades. (Table 1)

**Table 1:** Haemoglobin levels across pallor categories (observer 1, n = 390).

Site	Haemoglobin (g/dL)	pallor grading*				P values†		
		0	1	2	3	1 vs. 0	2 vs. 1	3 vs. 2
Conjunctiva	Mean	12.4	11.2	10.5	8.7	<0.01	0.01	<0.01
	Standard deviation	(2.1)	(2.4)	(2.3)	(3.1)			
	Number	89	162	98	35			
Tongue	Mean	12.3	11.4	9.3	7.2	<0.01	<0.01	0.05
	Standard deviation	(2.3)	(2.2)	(2.8)	(2.8)			
	Number	70	220	64	9			
Nailbed	Mean	11.9	11.5	9.6	8.5	0.12	<0.01	0.25
	Standard deviation	(2.4)	(2.3)	(2.6)	(2.8)			
	Number	130	130	106	9			
Palm	Mean	11.9	10.8	10.1	8.3	<0.01	0.07	0.06
	Standard deviation	(2.2)	(2.7)	(2.7)	(2.9)			
	Number	153	121	100	8			

*0 =  no pallor; 1 =  mild pallor; 2 =  moderate pallor; 3 =  severe pallor.

†All p values were determined using student's *t* test for difference between the means.

The prevalence of anaemia at haemoglobin cutoff point of 7 g/dL was 7%. Tongue pallor outperformed other pallor sites for ruling in moderate as well as severe anaemia: (LR 7.6 (1.9, 29.7) and 9.87 (2.81, 34.6)) respectively (Table 2). A clear gradient in point estimates of likelihood ratios was observed for all pallor sites, for detection of severe anaemia The Bayesian approach to diagnostic studies (pretest odds × LR  =  post-test odds) suggests that at haemoglobin cutoff point 9 g/dL, severe tongue pallor produced a probability of 44% (14, 79) while absence of tongue pallor virtually ruled out anaemia (LR, 0). At haemoglobin cutoff point 9 g/dL (prevalence of anaemia, 21%) severe tongue pallor increased the probability to 67% (30, 92) while absence of tongue pallor decreased the probability to 7% (2, 15). Palmer, conjunctival and nailbed pallor didn't generate LRs, powerful enough to confidently rule in or rule out anaemia at different haemoglobin thresholds.

**Table 2:** Likelihood ratios (LR) for pallor grades in diagnosing anaemia.

Site	Pallor	Haemoglobin – 7 g/dL	Haemoglobin – 9 g/dL	Haemoglobin – 12 g/dL
		Likelihood Ratios (95% CI)		
Conjunctiva	Severe	3.51 (0.76, 16.1)	4.42 (1.21, 16.1)	4.25 (0.53, 33.6)
	Moderate	2.32 (1.77, 3.04)	1.89 (1.45, 2.46)	1.86 (1.32, 2.64)
	Mild	1.34 (1.26, 1.42)	1.23 (1.11, 1.36)	1.37 (1.18, 1.58)
	No	–	0.38 (0.19, 0.74)	0.40 (0.28, 0.58)
Tongue	Severe	9.87 (2.81, 34.6)	7.60 (1.95, 29.7)	–
	Moderate	4.98 (3.61, 6.87)	3.70 (2.50, 5.42)	3.19 (1.74, 5.82)
	Mild	1.27 (1.20, 1.34)	1.21 (1.11, 1.32)	1.18 (1.05, 1.33)
	No	–	0.29 (0.12, 0.70)	0.53 (0.35, 0.81)
Nailbed	Severe	3.51 (0.76, 16.1)	4.42 (1.21, 16.1)	4.25 (0.53, 33.6)
	Moderate	2.59 (1.94, 3.46)	2.44 (1.85, 3.23)	2.86 (1.81, 4.51)
	Mild	1.35 (1.13, 1.60)	1.29 (1.12, 1.49)	1.48 (1.23, 1.79)
	No	0.39 (0.16, 0.98)	0.53 (0.34, 0.84)	0.52 (0.39, 0.68)
Palm	Severe	4.18 (0.88, 19.8)	5.94 (1.45, 24.4)	3.70 (0.46, 29.7)
	Moderate	2.20 (1.53, 3.18)	1.66 (1.20, 2.30)	1.93 (1.28, 2.91)
	Mild	1.55 (1.33, 1.81)	1.43 (1.23, 1.67)	1.47 (1.20, 1.81)
	No	0.25 (0.08, 0.74)	0.48 (0.31, 0.73)	0.60 (0.47, 0.76)

CI - Confidence Intervals.

**Figure 2:**
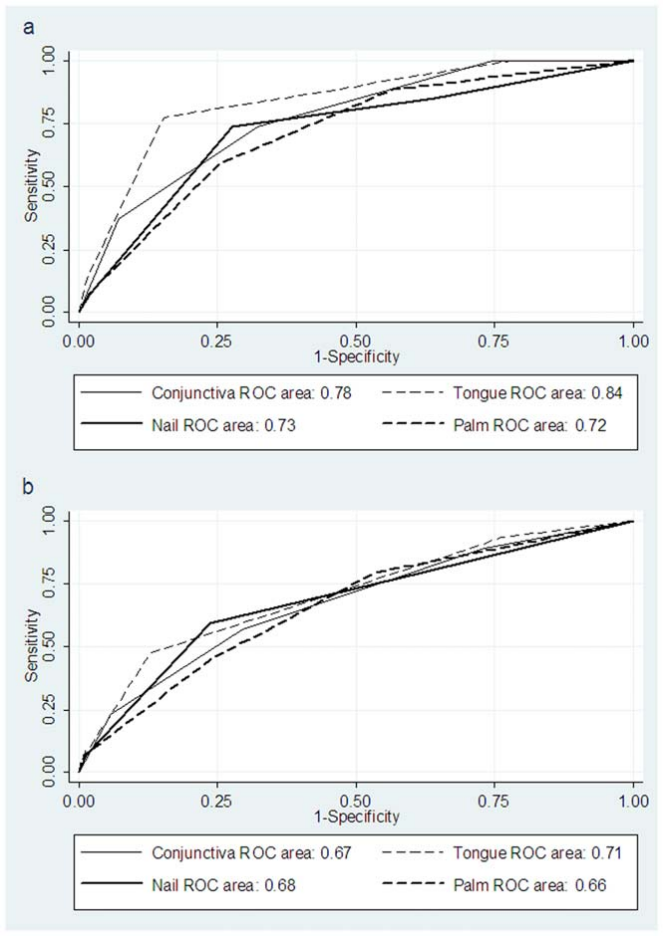
Receiver Operating Characteristics (ROC) curves for all clinical sites for detection of anaemia at haemoglobin cutoff points (a) 7 g/dL and (b) 9 g/dL.

Receiver operating characteristic curves showed that at haemoglobin cutoff point of 9 g/dL, none of the pallor sites was superior to the other (ROC area ranging from 0.66 to 0.70). At haemoglobin cutoff point of 7 g/dL, although tongue (ROC area, 0.84(0.77 to 0.90)) was superior to conjunctiva (ROC area 0.77(0.69, 0.85)), the difference was not statistically significant (p = 0.06); nailbed and palmer pallor sites did not perform as well. (Figure 2)

Data on agreement between the two observers was available for 128 patients. The agreement was poor for all anatomical sites. The kappa statistic (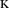) for the conjunctival pallor was 0.07, and that for tongue, finger nailbed and tongue pallor was 0.19, 0.18, and 0.09 respectively. (Table 3).

**Table 3:** Interobserver variability in assessment of anaemia (n = 128).

Observer 1 vs. Observer 2	Percent agreement	Kappa value	95% CI
Conjunctival pallor	35.20	0.07	0.04, 0.14
Tongue pallor	43.12	0.19	0.09, 0.28
Nailbed pallor	41.46	0.18	0.08, 0.27
Palmer pallor	38.52	0.09	0.02, 0.20

## Discussion

Our main finding is that presence of pallor can modestly raise the probability of severe anaemia while its absence can rule out severe anaemia. Neither presence nor absence of pallor, regardless of its severity, can accurately rule in or rule out moderate anaemia.

The published literature about accuracy of clinical signs has reported a wide range of sensitivity and specificity estimates varying from 19% to 70% and 70% to 100% respectively [2], [4], [5], [6], [8]. Nardone et al [6] found that at haematocrit cut off point of 0.30, conjunctival pallor (LR+2.33), and pallor of face (LR+2.52) were poor predictors of anaemia. Even combination of all four anatomical sites (conjunctiva, tongue, palm and nailbed) has not made pallor a more informative test [9]: 76% sensitivity and 63% specificity at haemoglobin threshold of 5 g/dL and 59% and 64% respectively at haemoglobin threshold of 7 g/dL.

In the present study, we evaluated the accuracy of pallor by examining four pallor grades- none, mild, moderate and severe (instead of present or absent): a grading commonly used by physicians at the bedside. This approach is closer to clinical practice, and we could use multilevel likelihood ratios in diagnostic accuracy estimates. According to a mnemonic suggested by McGee [10], likelihood ratios 2, 5 and 10 increases the probability of target disorder by about 15%, 30% and 45% respectively. Our findings suggest that at the haemoglobin cutoff point of 7 g/dL, absence of conjunctival pallor and tongue pallor completely ruled out the probability of haemoglobin <7 g/dL. However, at haemoglobin cutoff points of 9 g/dL, no physical site generated LRs, large enough to rule in or small enough to rule out anaemia.

Despite severe tongue pallor having a large likelihood ratio of 9.87, it did not result in a large and meaningful shift in the post-test probability of severe anaemia in our study (from 7% to 52%). This was so, not because the test was weak, but because the pretest probability of severe anaemia in our patients was low [11]. Our results indicate that severe tongue pallor may be used as a screening test to order haemoglobin in resource-limited settings. Severely pale tongue, as a screening test, has several virtues; it costs nothing, is painless, and can be easily elicited even in crowded out-patient departments in just a few seconds. A pink tongue can reassure the physicians that haemoglobin test may not be necessary.

In contrast to previous studies that suggested that conjunctivae [6]; palms or nailbeds [12] were better sites to detect pallor, in our study, tongue outperformed other sites. Our observations concur with a previous study [6] that suggested that examination of nailbeds is inferior to all other pallor sites. In our study, examination of conjunctivae, nailbeds, or palms failed to identify several severely anaemic patients; conversely several patients with normal haemoglobin were adjudged anaemic by the pallor assessment. We cannot explain why we failed to pick up pale conjunctivae even in patients with severe anaemia, an anomaly also reported previously [2].

Our kappa scores of interobserver agreement between paired observers concur with two those from previous studies [4], [6] in which scores ranged from 0.16 to 0.51[6]; and from 0.23–0.47 [4]. One study reported high kappa scores [2] (0.75 and 0.54), possibly because the researchers standardized their physical assessments before they began the study.

According to Moons [13], diagnostic process is multivariable and sequential and therefore, the multivariate analysis should be a focus of diagnostic data analysis. In our study, even as the observers assessed pallor in a particular order (conjunctiva, tongue, palm and nailbed) we did not obtain a gradient in terms of increasing ROC areas in the same order. Thus it is unlikely that assessment of pallor at one site was influencing decision-making at another.

Comparison of our results with those of other studies must be made with caution because our patients were aged 12 years and above; we examined them in a hospital setting and used electronic cell counter to measure haemoglobin. By contrast, most studies on accuracy of pallor to detect anaemia are community based [9], [14], [15], [16], [17], have evaluated children [12], [18], [19], [20], [21], [22], [23], and used colour comparison techniques to measure haemoglobin [18], [24].

Nardone et al. argued [6], and we agree with them that pallor as a screening test should reassure the physicians that haemoglobin test is not necessary when pallor is absent, than be of help for ruling in anaemia when pallor is present. Also, physicians use pallor to conform or rule out moderate or severe anaemia, and not mild anaemia [4]. Therefore, we evaluated the diagnostic accuracy of pallor at haemoglobin cut off points of 7 g/dL and 9 g/dL. Although pallor has been found to be more accurate in detecting anaemia when haemoglobin cut off values are lower [6], Group et al [4] found that even in patients with the lowest haemoglobin concentrations, the post test probability of pallor to detect anaemia did not exceed 75%, because patients judged severely pale do not necessarily have severe anaemia.

Our study has several strengths. Our patients represent typical rural Indian patients. By including the whole spectrum of consecutive inpatients and outpatients in our study we avoided the spectrum and selection bias. We made a blind and independent comparison between the assessment of pallor (diagnostic test) and measurement of haemoglobin (the reference standard). We used an electronic cell counter to measure haemoglobin, which has less variability than haemoglobin levels measured by portal HemoCue systems [24]. We used multilevel likelihood ratios for evaluating the diagnostic accuracy of pallor. We evaluated the diagnostic accuracy of combination of physical signs, a method used by physicians at the bedside. The major limitation of our study is that the results of the study cannot be generalised to the community settings and to children. Second, the study researchers were not explicitly trained for accessing pallor, thus the physical examination was not standardised. We did this deliberately and designed our study to reflect actual clinical practice and to enable evaluation of the diagnostic accuracy of pallor as it is currently used in busy outpatient and inpatient settings. Also, the small size of one of the subgroups led to imprecise estimates of likelihood ratios.

In conclusion, health professionals may use tongue pallor for ruling out and modestly ruling in severe anaemia. Patients with suspected severe anaemia need to have their haemoglobin measured.
